# Postpandemic COVID-19 Vaccination Willingness Among Physicians and Medical Trainees in China: Cross-Sectional Study

**DOI:** 10.2196/88931

**Published:** 2026-10-06

**Authors:** Yuxian Mu, Yeming Wang, Xiaoying Gu, Lianhan Shang, Wuqiang Che, Weixia Xuan, Xiaoyu Cao, Chao Zhang, Xiaolin Zhou, Shengnan Sun, Rui Zhao, Anli Liu, Jianyu Kuang, Yuqi Guo, Jinying Mo, Bin Cao

**Affiliations:** 1 China-Japan Friendship Hospital (Institute of Clinical Medical Sciences), Chinese Academy of Medical Sciences & Peking Union Medical College, Beijing, 100029, China, 86 010-8420-5566; 2 National Center for Respiratory Medicine, State Key Laboratory of Respiratory Health and Multimorbidity, National Clinical Research Center for Respiratory Diseases, Institute of Respiratory Medicine, Chinese Academy of Medical Sciences & Peking Union Medical College, Department of Pulmonary and Critical Care Medicine, Center of Respiratory Medicine, China-Japan Friendship Hospital, Beijing, China; 3 Department of Clinical Research and Data Management, Center of Respiratory Medicine, China-Japan Friendship Hospital, Beijing, 100029, China; 4 Department of Cardiology, China-Japan Friendship Hospital, Beijing, 100029, China; 5 Department of Pulmonary and Critical Care Medicine, Henan Provincial People's Hospital, Zhengzhou, Henan, China; 6 Department of Pulmonary and Critical Care Medicine, The Second People's Hospital of Fuyang City; Fuyang Infectious Disease Clinical College, Anhui Medical University, 1088 Yinghe West Road, Fuyang, Anhui, 236015, China; 7 Department of Respiratory and Critical Care Medicine, Affiliated Central People’s Hospital, China Three Gorges University, Yichang, Hubei, China; 8 Department of Infectious Diseases, Yichang Central People's Hospital, The First College of Clinical Medical Science, China Three Gorges University, Yichang, Hubei, 443001, China; 9 The Sixth People's Hospital of Shenyang, Shenyang, Liaoning, China; 10 Department of Pulmonary and Critical Care Medicine, The First People's Hospital of Longquanyi District Chengdu, Chengdu, Sichuan, China; 11 Department of Infectious Diseases, Longgang Central Hospital, Shenzhen, Guangdong, China; 12 Intensive Care Unit, Xi'an Chest Hospital, Xi'an, Shaanxi, China; 13 Tsinghua University-Peking University Joint Center for Life Sciences, Tsinghua University, Beijing, 100084, China

**Keywords:** COVID-19 vaccination, vaccine hesitancy, annual vaccination, physicians, medical trainees

## Abstract

**Background:**

As SARS-CoV-2 continues to evolve and vaccine-induced protection wanes over time, future annual or periodic COVID-19 vaccination may become important for long-term disease control. China’s adjustment of national COVID-19 response policies provided an opportunity to assess vaccination attitudes after extensive prior vaccination and widespread SARS-CoV-2 exposure. Evidence remains limited on physicians’ and medical trainees’ willingness to receive future COVID-19 vaccination and to support COVID-19 vaccination for older adults and young children.

**Objective:**

This study aimed to evaluate willingness to receive future annual COVID-19 vaccination among physicians and medical trainees in China, identify factors associated with hesitancy toward future vaccination, and assess willingness to support COVID-19 vaccination for older adults and young children.

**Methods:**

A nationwide, multicenter cross-sectional survey was conducted from February to April 2023, shortly after China adjusted its national COVID-19 response policies. Physicians and medical trainees were recruited from 21 public hospitals across 7 regions of China using prespecified recruitment targets for each hospital and career-stage group. Participants completed a confidential, self-administered electronic questionnaire. Modified Poisson regression models with robust SEs were used to estimate adjusted prevalence ratios (aPRs) and 95% CIs.

**Results:**

The analysis included 3169 participants comprising 2877 physicians (90.8%) and 292 medical trainees (9.2%). Overall, 1899 participants (59.9%) were willing to receive future annual COVID-19 vaccination, although 3074 (97%) had previously received at least 1 dose of a COVID-19 vaccine. Among previously vaccinated participants, 2862 (93.1%) had received an inactivated vaccine. Overall, 2857 participants (90.2%) were willing to support COVID-19 vaccination for older adults, and 2320 (73.2%) were willing to support vaccination for young children. Lack of willingness to receive influenza vaccination was strongly associated with hesitancy toward future annual COVID-19 vaccination (aPR 4.11; 95% CI 3.71‐4.55), as was no prior COVID-19 vaccination (aPR 1.27; 95% CI 1.11‐1.46). Participants with hesitancy toward future annual COVID-19 vaccination were more likely to report hesitancy to support vaccination for older adults (aPR 5.13; 95% CI 3.95‐6.66) and young children (aPR 2.88; 95% CI 2.54‐3.27). Among participants hesitant about future annual vaccination, the most commonly reported concerns were vaccine effectiveness (674/1270, 53.1%), vaccine safety (424/1270, 33.4%), and insufficient disclosure of clinical trial data (368/1270, 29%).

**Conclusions:**

Hesitancy toward future annual COVID-19 vaccination was substantial among physicians and medical trainees in China despite near-universal prior vaccination. The most commonly reported concerns were related to vaccine effectiveness, vaccine safety, and clinical data transparency. Clear and timely communication of vaccine evidence to clinical professionals may support future routine or periodic COVID-19 vaccination strategies and strengthen vaccination support for older adults and young children.

## Introduction

Vaccine hesitancy remains a major public health challenge. The World Health Organization identified vaccine hesitancy as one of the major threats to global health, and the Strategic Advisory Group of Experts Working Group defined vaccine hesitancy as a delay in acceptance or refusal of vaccination despite the availability of vaccination services [1,2]. Vaccine willingness is context specific and may vary across time, place, and vaccine type, reflecting perceived disease risk, confidence in vaccine safety and effectiveness, access-related constraints, information seeking and deliberation, and broader social or policy contexts [2-4]. These issues are particularly relevant for vaccines that may require repeated, annual, or risk-based use.

COVID-19 vaccination illustrates this challenge. COVID-19 vaccines have substantially reduced severe disease and mortality worldwide [5,6]. However, SARS-CoV-2 continues to evolve, and vaccine-induced protection, particularly against infection and symptomatic disease, may wane over time [7,8]. As COVID-19 shifts from emergency pandemic response to longer-term disease management, the relevant public health question has moved from primary vaccine acceptance to willingness to receive future annual or periodic vaccination [9]. Physicians and medical trainees are a key population in this context because they are both potential vaccine recipients and trusted sources of vaccine information for patients and the public [1,10]. Their confidence in vaccine effectiveness, safety, and clinical evidence may influence both their own vaccination decisions and their willingness to support vaccination for others, including older adults and young children.

The early 2023 period in China provided a useful setting for examining willingness to continue COVID-19 vaccination after large-scale prior vaccination and changing perceived disease risk. By late 2022, more than 3.4 billion COVID-19 vaccine doses had been administered nationwide [11], and COVID-19 vaccines available in China included domestically produced inactivated vaccines, adenoviral-vector vaccines, and recombinant protein vaccines [12-14]. Shortly before we conducted this survey, China adjusted its national COVID-19 response policies, marking a shift from emergency containment toward longer-term management of SARS-CoV-2 infection [15]. We therefore conducted a nationwide cross-sectional survey in 21 public hospitals across 7 regions of China to evaluate willingness to receive future annual COVID-19 vaccination among physicians and medical trainees, identify factors associated with hesitancy, assess willingness to support COVID-19 vaccination for older adults and young children, and assess willingness to receive influenza vaccination as a comparative reference for periodic vaccination.

## Methods

### Study Design and Setting

This was a nationwide, multicenter, cross-sectional questionnaire survey conducted from February to April 2023 in 21 public hospitals across 7 geographic regions of China: North China, Northeast China, East China, Central China, Northwest China, Southwest China, and South China. The participating hospitals were located in 15 provinces, municipalities, and autonomous regions.

A pilot survey was conducted in December 2022 among 108 physicians and medical trainees to assess questionnaire clarity and the feasibility of electronically administering the survey. The pilot confirmed the feasibility of the survey procedures, informed refinement of the questionnaire, and provided data for sample-size planning. Pilot participants were not included in the final analysis.

### Questionnaire Development

The questionnaire was developed based on literature review, expert consultation, and pilot testing. Content validity was assessed through expert review using the content validity ratio (CVR). Internal consistency was evaluated using the Cronbach α, with values of 0.65 for the confidence domain and 0.68 for the complacency domain. The questionnaire can be found in Multimedia Appendix 1.

### Participants and Recruitment

Eligible participants were physicians and medical trainees working or training at the participating hospitals during the study period. The survey focused on the physician workforce across career stages because physicians’ vaccination attitudes may influence patient counseling and vaccination-related clinical decisions, while medical trainees represent the earliest stage of physician training. Recruitment was organized at the hospital level using prespecified target numbers for each hospital and career-stage group, including medical trainees who had not begun standardized residency training, residents or physicians undergoing standardized residency training, attending physicians, associate chief physicians, and chief physicians.

At each hospital, 2 designated investigators coordinated recruitment and questionnaire distribution. To ensure broad representation across clinical departments, investigators sought to include participants from departments directly involved in COVID-19 care, including pulmonary and critical care medicine, emergency medicine, infectious diseases, and intensive care medicine, as well as other departments caring for patients at increased risk of severe COVID-19. Participants with and without experience treating patients with COVID-19 were actively recruited. Representation across clinical departments and experience treating patients with COVID-19 were recruitment targets rather than prespecified quotas.

The final questionnaire was self-administered electronically through Wenjuanxing (Changsha Ranxing Information Technology Co Ltd), and the survey link was distributed through direct electronic invitations and department-level WeChat (Tencent Holdings Limited) groups. Recruitment continued until the prespecified target numbers for each hospital and career-stage group were reached. Because the questionnaire link was distributed through both direct electronic invitations and department-level WeChat groups, the exact number of individuals who received or viewed the invitation could not be precisely determined; therefore, a response rate could not be calculated. The survey platform allowed only 1 submission per WeChat ID to reduce duplicate responses.

The sample size was estimated using PASS version 21.0 (NCSS, LLC) based on pilot survey data. The calculation incorporated a design effect of 1.2 to account for potential clustering within hospitals. Each hospital aimed to recruit approximately 150 eligible participants, yielding a target sample size of approximately 3150 participants across 21 hospitals. All submitted questionnaires with complete outcome and covariate information required for the main analyses were included in the final analytic dataset.

### Study Outcomes and Measures

The primary outcome was hesitancy toward future annual COVID-19 vaccination, assessed using the following question: “Would you proactively receive the COVID-19 vaccine every year in the future?” Participants who answered “no” were classified as having hesitancy toward future annual COVID-19 vaccination and were asked to select reasons for hesitancy.

Secondary outcomes were hesitancy to support COVID-19 vaccination for older adults and for young children in participants’ families. These outcomes were assessed using the following questions: “If older adults in your family aged over 65 years have no clear contraindications, would you support their COVID-19 vaccination?” and “If there are children younger than 6 years in your family, would you support their COVID-19 vaccination?” Participants who answered “no” were classified as having hesitancy to support COVID-19 vaccination for the corresponding group.

The questionnaire collected information on age, education level, geographic region, hospital classification, clinical department, career stage, SARS-CoV-2 infection history, experience treating patients with COVID-19, prior COVID-19 vaccination, type of COVID-19 vaccine received, willingness to receive influenza vaccination, and selected perceptions related to COVID-19. Data on sex and income were not collected in this survey.

Regions were categorized as low, middle, or high according to the Human Development Index (HDI) of the province-level administrative regions in which the participating hospitals were located. Hospitals were classified as top grade A tertiary hospitals, other grade A tertiary hospitals, and nontertiary or other hospitals based on China’s hospital classification system and prespecified hospital ranking criteria. Clinical departments were categorized as COVID-19–related internal medicine departments, other internal medicine departments, surgical departments, or other departments. Educational attainment was categorized as doctoral degree, master’s degree, and college degree or lower.

### Ethical Considerations

The study protocol was reviewed and approved by the Ethics Review Committee for Biomedical Research Involving Human Subjects of the Chinese Academy of Medical Sciences and Peking Union Medical College (approval number CAMS&PUMC-IEC-2023‐030). This study was conducted in accordance with the Declaration of Helsinki. Before completing the questionnaire, all participants provided electronic informed consent (Multimedia Appendix 2).

The questionnaire did not collect participants’ names, national identification numbers, home addresses, or other identifying information for research analysis. Mobile phone numbers were collected solely for distributing participant compensation and were excluded from the analytic dataset. The WeChat ID restriction was used only to prevent duplicate submissions, and WeChat identifiers were not included in the analytic dataset. Survey data were stored in a password-protected database accessible only to authorized study investigators. Participants received CNY 50 (US $1=CNY 6.87 as of March 1, 2023) after completing the questionnaire.

### Statistical Analysis

Participant characteristics were summarized as frequencies and percentages and compared according to hesitancy toward future annual COVID-19 vaccination (Table 1). Group comparisons were conducted according to expected cell counts. The Pearson *χ*^2^ test was used when all expected cell counts were 5 or greater, and the Fisher exact test was used when any expected cell count was less than 5. All tests were 2-sided, and a *P* value <.05 was considered statistically significant.

**Table 1.** Characteristics of participants stratified by hesitancy or willingness toward future annual COVID-19 vaccination.

Characteristic	Participants (N=3169), n (%)	Hesitancy (N=1270), n (%)	Willingness (N=1899), n (%)	*P* value
Age, years				.001
20‐29	1125 (35.5)	501 (39.4)	624 (32.9)	
30‐39	1337 (42.2)	491 (38.7)	846 (44.5)	
40‐49	514 (16.2)	203 (16.0)	311 (16.4)	
≥50	193 (6.1)	75 (5.9)	118 (6.2)	
Education level				<.001
College or lower	1305 (41.2)	434 (34.2)	871 (45.9)	
Master’s degree	1505 (47.5)	657 (51.7)	848 (44.7)	
Doctoral degree	359 (11.3)	179 (14.1)	180 (9.5)	
HDI^a^ level				.007
High HDI	919 (29.0)	389 (30.6)	530 (27.9)	
Middle HDI	1472 (46.4)	606 (47.7)	866 (45.6)	
Low HDI	778 (24.6)	275 (21.7)	503 (26.5)	
Hospital classification				<.001
Top grade A tertiary hospital	1202 (37.9)	561 (44.2)	641 (33.8)	
Other grade A tertiary hospital	1195 (37.7)	439 (34.6)	756 (39.8)	
Nontertiary or other hospital	772 (24.4)	270 (21.3)	502 (26.4)	
Clinical department				.26
COVID-19–related internal medicine departments	1080 (34.1)	427 (33.6)	653 (34.4)	
Other internal medicine departments	1228 (38.8)	489 (38.5)	739 (38.9)	
Surgical departments	420 (13.3)	186 (14.6)	234 (12.3)	
Other departments	441 (13.9)	168 (13.2)	273 (14.4)	
Career stage				.005
Medical trainee	292 (9.2)	110 (8.7)	182 (9.6)	
Resident	1173 (37.0)	517 (40.7)	656 (34.5)	
Attending physician	1010 (31.9)	375 (29.5)	635 (33.4)	
Associate chief/chief physician	694 (21.9)	268 (21.1)	426 (22.4)	
SARS-CoV-2 infection history				.30
No	215 (6.8)	79 (6.2)	136 (7.2)	
Yes	2954 (93.2)	1191 (93.8)	1763 (92.8)	
Experience treating patients with COVID-19				.08
No	601 (19.0)	260 (20.5)	341 (18.0)	
Yes	2568 (81.0)	1010 (79.5)	1558 (82.0)	
Prior COVID-19 vaccination				<.001
Yes	3074 (97.0)	1210 (95.3)	1864 (98.2)	
No	95 (3.0)	60 (4.7)	35 (1.8)	
Willingness to receive influenza vaccination				<.001
Yes	1932 (61.0)	345 (27.2)	1587 (83.6)	
No	1237 (39.0)	925 (72.8)	312 (16.4)	

a HDI: Human Development Index.

Because the primary and secondary outcomes were common, modified Poisson regression models with robust SEs were used to estimate unadjusted prevalence ratios (PRs), adjusted prevalence ratios (aPRs), and 95% CIs. All multivariable models were adjusted for age, education level, HDI level, hospital classification, clinical department, career stage, SARS-CoV-2 infection history, experience treating patients with COVID-19, and prior COVID-19 vaccination. The model for hesitancy toward future annual COVID-19 vaccination additionally included willingness to receive influenza vaccination, whereas the models for hesitancy to support vaccination for older adults and young children included participants’ own hesitancy toward future annual COVID-19 vaccination. Full univariable and multivariable modified Poisson regression results are reported in Table S4 in Multimedia Appendix 3. All analyses were performed using R software (version 4.3.2; R Foundation for Statistical Computing).

## Results

### Participant Characteristics

Among the 3169 participants included in the final analysis, the largest age group was 30 to 39 years (1337/3169, 42.2%), followed by 20 to 29 years (1125/3169, 35.5%). Overall, 1173 participants (37.01%) were residents, 1010 (31.87%) were attending physicians, 694 (21.9%) were associate chief or chief physicians, and 292 (9.2%) were medical trainees. Most participants reported a history of SARS-CoV-2 infection (2954/3169, 93.2%) and experience treating patients with COVID-19 (2568/3169, 81%).

Participant characteristics stratified by hesitancy toward future annual COVID-19 vaccination are shown in Table 1. Participants with and without hesitancy differed by age (*P*=.001), education level (*P*<.001), HDI level (*P*=.007), hospital classification (*P*<.001), career stage (*P*=.005), prior COVID-19 vaccination (*P*<.001), and willingness to receive influenza vaccination (*P*<.001). No significant differences were observed for clinical department, SARS-CoV-2 infection history, or experience treating patients with COVID-19.

### Future Annual COVID-19 Vaccination Willingness and Associated Factors

Overall, 1899 of 3169 participants (59.9%) were willing to receive future annual COVID-19 vaccination, whereas 1270 (40.1%) were classified as having hesitancy toward future annual COVID-19 vaccination. Most participants had previously received at least 1 dose of a COVID-19 vaccine (3074/3169, 97%). Among previously vaccinated participants, 2862 of 3074 (93.1%) had received an inactivated vaccine, and 1864 (60.6%) were willing to receive future annual COVID-19 vaccination. Vaccine types previously received and preferred vaccine types among participants willing to receive future COVID-19 vaccination are shown in Figure S1 of Multimedia Appendix 3.

In addition, 1932 participants (61%) were willing to receive influenza vaccination. Among participants willing to receive influenza vaccination, 1587 of 1932 (82.1%) were also willing to receive future annual COVID-19 vaccination compared with 312 of 1237 (25.2%) among those unwilling to receive influenza vaccination.

In adjusted analyses, unwillingness to receive influenza vaccination was strongly associated with a higher prevalence of hesitancy toward future annual COVID-19 vaccination (aPR 4.11; 95% CI 3.71‐4.55). Not having previously received COVID-19 vaccination was also associated with a higher prevalence of hesitancy (aPR 1.27; 95% CI 1.11‐1.46). Compared with participants with college-level education or lower, those with a master’s degree (aPR 1.11; 95% CI 1.02‐1.21) or doctoral degree (aPR 1.20; 95% CI 1.07‐1.36) had a higher prevalence of hesitancy. Compared with participants from top grade A tertiary hospitals, those from other grade A tertiary hospitals (aPR 0.89; 95% CI 0.81‐0.97) and nontertiary or other hospitals (aPR 0.81; 95% CI 0.72‐0.90) had a lower prevalence of hesitancy (Figure 1). Full results for this outcome are shown in Table S4A of Multimedia Appendix 3.

**Figure 1.**
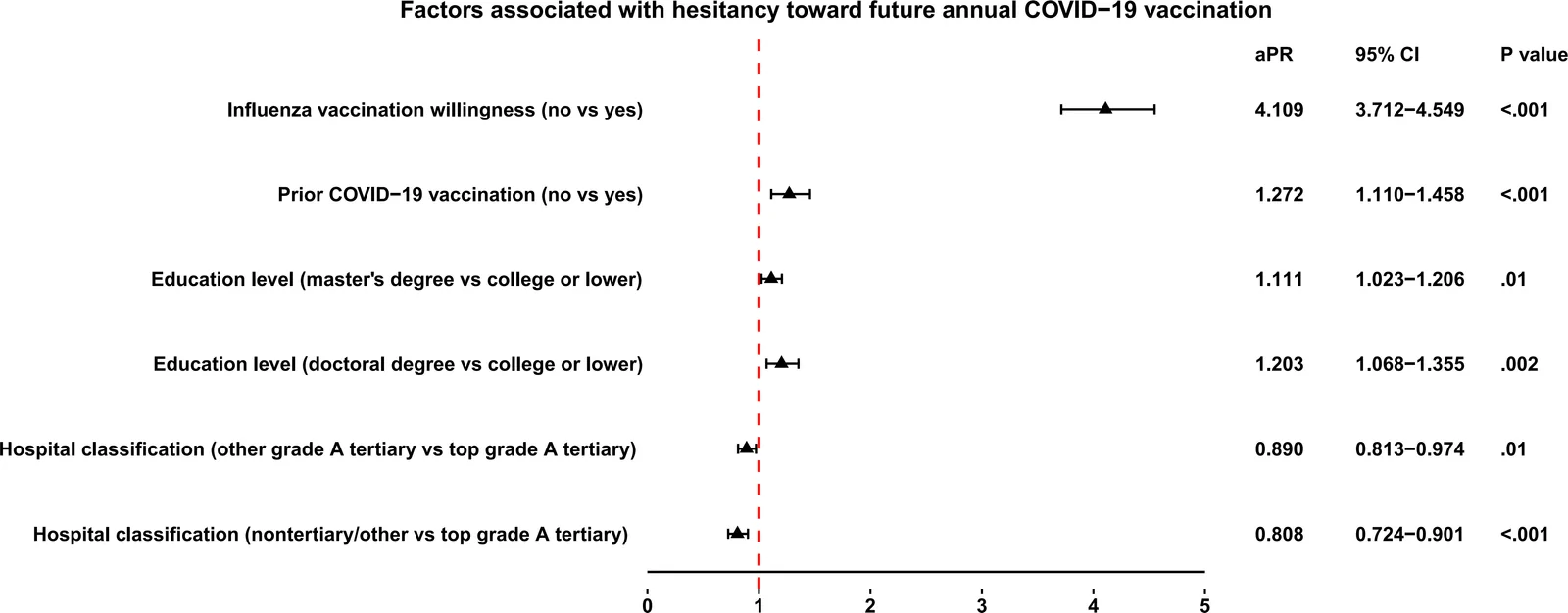
Factors associated with hesitancy toward future annual COVID-19 vaccination among physicians and medical trainees. Adjusted prevalence ratios (aPRs) and 95% CIs were estimated using modified Poisson regression models with robust SEs. Full model results are shown in Table S4A of Multimedia Appendix 3.

### Support for COVID-19 Vaccination for Older Adults and Young Children

Overall, 2857 of 3169 participants (90.2%) were willing to support COVID-19 vaccination for adults aged over 65 years without contraindications, and 2320 (73.2%) were willing to support vaccination for children younger than 6 years. For both target groups, the proportion willing to support vaccination was lower among participants with hesitancy toward future annual COVID-19 vaccination. Among hesitant participants, 1026 of 1270 (80.8%) supported vaccination for older adults and 705 (55.5%) supported vaccination for young children compared with 1831 of 1899 (96.4%) and 1615 (85%), respectively, among those willing to receive future annual COVID-19 vaccination.

In adjusted analyses, participants who were hesitant about receiving future annual COVID-19 vaccination themselves had a higher prevalence of hesitancy to support COVID-19 vaccination for older adults (aPR 5.13; 95% CI 3.95‐6.66). Hesitancy to support vaccination for older adults was also higher among participants from other internal medicine departments (aPR 1.36; 95% CI 1.05‐1.78) and surgical departments (aPR 1.69; 95% CI 1.24‐2.31) compared with those from COVID-19–related internal medicine departments. Attending physicians (aPR 0.57; 95% CI 0.35‐0.93), associate chief or chief physicians (aPR 0.48; 95% CI 0.26‐0.89), and participants with a history of SARS-CoV-2 infection (aPR 0.71; 95% CI 0.51‐0.98) had a lower prevalence of hesitancy to support vaccination for older adults (Figure 2A). Full results for this outcome are shown in Table S4B of Multimedia Appendix 3.

Similar associations were observed for hesitancy to support vaccination for young children. Participants who were hesitant about receiving future annual COVID-19 vaccination themselves had a higher prevalence of hesitancy to support COVID-19 vaccination for young children (aPR 2.88; 95% CI 2.54‐3.27). Not having previously received COVID-19 vaccination was also associated with a higher prevalence of hesitancy to support vaccination for young children (aPR 1.42; 95% CI 1.16‐1.74). Compared with participants aged 20 to 29 years, those aged 40 to 49 years (aPR 1.39; 95% CI 1.07‐1.79) and 50 years or older (aPR 1.52; 95% CI 1.13‐2.06) had a higher prevalence of hesitancy. Participants from middle-HDI regions (aPR 0.82; 95% CI 0.71‐0.94), from low-HDI regions (aPR 0.75; 95% CI 0.63‐0.89), and with a history of SARS-CoV-2 infection (aPR 0.82; 95% CI 0.67‐0.99) had a lower prevalence of hesitancy to support vaccination for young children (Figure 2B). Full results for this outcome are shown in Table S4C of Multimedia Appendix 3.

**Figure 2.**
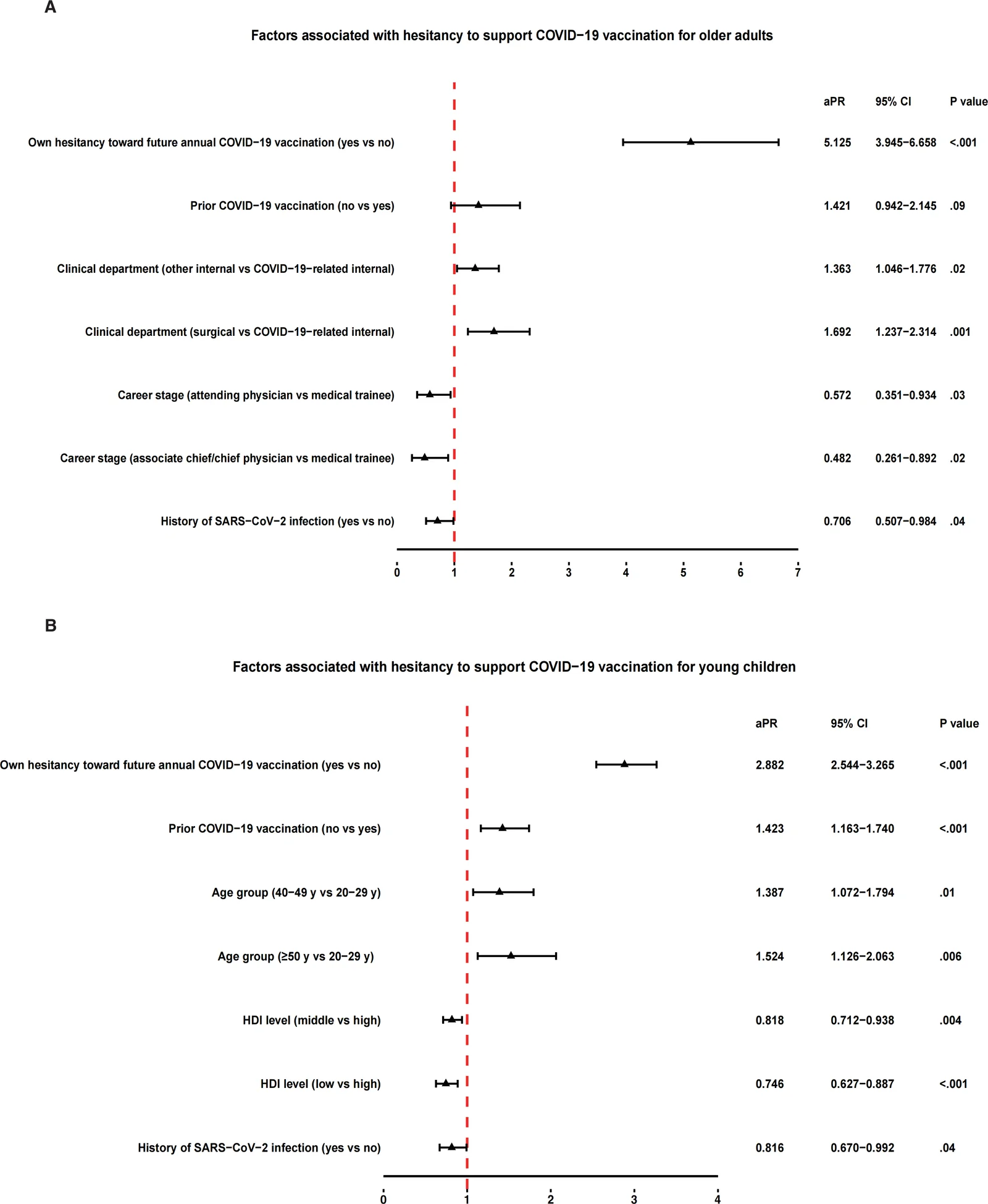
Factors associated with hesitancy to support COVID-19 vaccination for older adults aged over 65 years without clear contraindications (A) and children aged less than 6 years (B). Adjusted prevalence ratios (aPRs) and 95% CIs were estimated using modified Poisson regression models with robust standard errors. The vertical dashed line indicates an aPR of 1. Full model results are shown in Table S4B for older adults and Table S4C (Multimedia Appendix 3) for young children.

### Reported Reasons for Vaccine Hesitancy

Among participants with hesitancy toward future annual COVID-19 vaccination, the most frequently reported reasons were concerns about vaccine effectiveness (674/1270, 53.1%), concerns about vaccine safety (424/1270, 33.4%), and insufficient disclosure of domestic clinical trial data (368/1270, 29%). Additional responses included perceiving vaccination as unnecessary because they were in good health (235/1270, 18.5%), other reasons (221/1270, 17.4%), fear of injection (115/1270, 9.1%), and concerns about underlying disease or contraindications to vaccination (73/1270, 5.7%) (Figure 3A).

**Figure 3.**
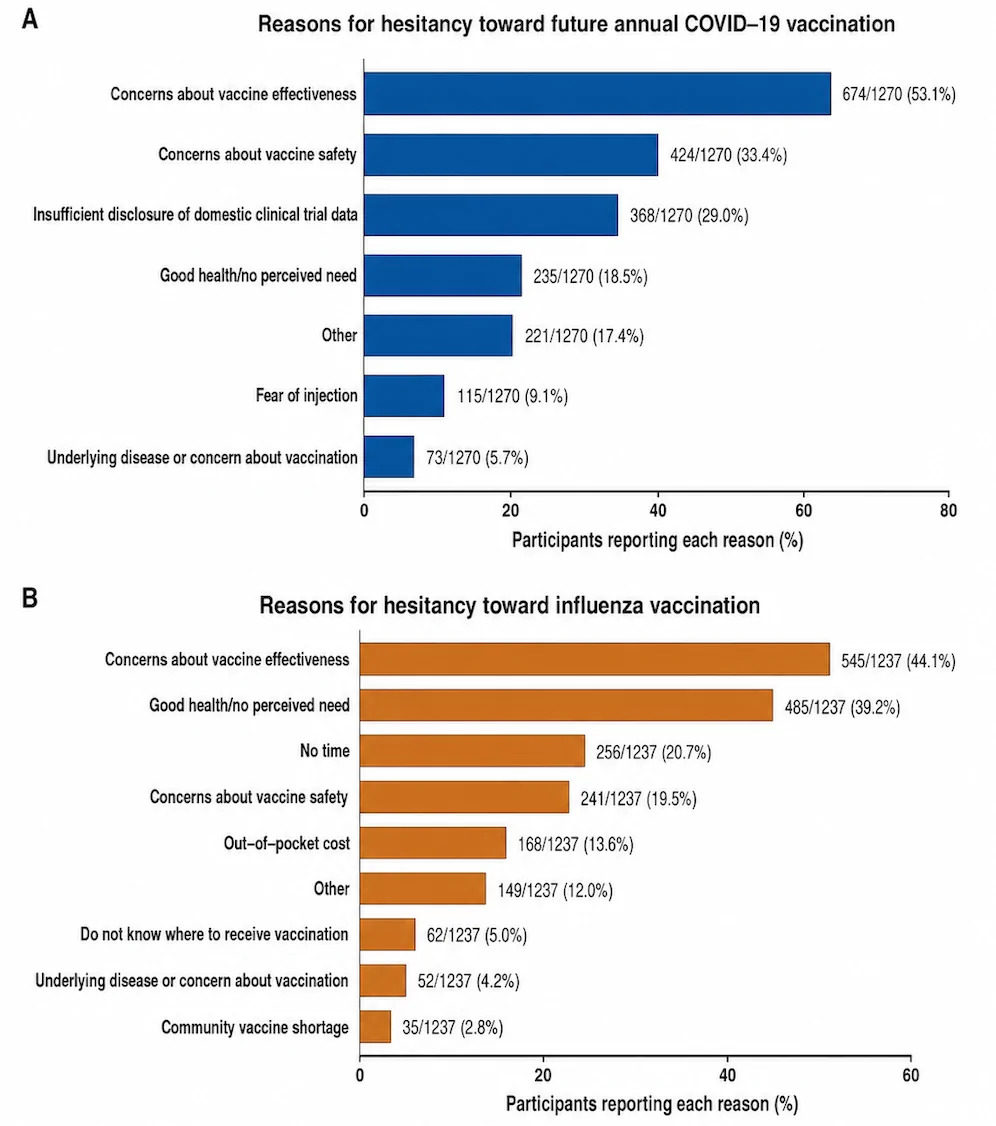
Reasons for hesitancy toward periodic COVID-19 and influenza vaccination among physicians and medical trainees.

Among participants with influenza vaccination hesitancy, the most frequently reported reasons were concerns about vaccine effectiveness (545/1237, 44.1%), perception of vaccination as unnecessary because they were in good health (485/1237, 39.2%), lack of time (256/1237, 20.7%), and concerns about vaccine safety (241/1237, 19.5%). Additional responses included out-of-pocket cost (168/1237, 13.6%), other reasons (149/1237, 12%), not knowing where to receive vaccination (62/1237, 5%), concerns about underlying disease or contraindications to vaccination (52/1237, 4.2%), and community vaccine shortage (35/1237, 2.8%) (Figure 3B).

## Discussion

This nationwide, multicenter, cross-sectional survey, conducted during the early 2023 transition from emergency COVID-19 response to longer-term disease management, showed a marked divergence between prior COVID-19 vaccination uptake and willingness to receive future annual vaccination among physicians and medical trainees in China. Despite near-universal prior vaccination in 3169 participants, 1899 (59.9%) participants were willing to receive future annual vaccination, a proportion similar to the 1932 (61%) willing to receive influenza vaccination. Participants’ own hesitancy was also associated with hesitancy to support COVID-19 vaccination for older adults and young children. The leading concerns involved vaccine effectiveness, vaccine safety, and clinical trial data transparency.

This divergence suggests that high prior COVID-19 vaccination uptake should not be assumed to indicate sustained willingness to receive repeated or periodic vaccination. Because vaccine hesitancy is context specific and may vary by time, place, and vaccine type [2,4,16], this pattern should not be interpreted simply as general vaccine refusal. Rather, it highlights the importance of distinguishing initial vaccine uptake during an emergency response from willingness to receive repeated vaccination in the postemergency phase of COVID-19 management. This distinction is particularly relevant to the study period, when China had recently adjusted its national COVID-19 response policies and the World Health Organization subsequently declared that COVID-19 was no longer a Public Health Emergency of International Concern [15,17].

Concerns about vaccine effectiveness, vaccine safety, and transparency of clinical trial data have also been reported among health care workers [18-20]. In this professional population, these concerns suggest that generic reassurance may be insufficient. The higher prevalence of hesitancy among participants with a master’s or doctoral degree and among those from top grade A tertiary hospitals further suggests that hesitancy may not simply reflect a lack of knowledge or general vaccine refusal. Rather, it may be partly evidence sensitive: physicians and medical trainees may question repeated vaccination when updated evidence is perceived as incomplete or insufficiently transparent [2,3,21].

The association between participants’ own vaccination hesitancy and their support for vaccination among older adults and young children is important because physicians and medical trainees may influence vaccination decisions beyond their personal vaccination behavior; health care providers’ vaccine perceptions and communication can shape patient receptivity and vaccine uptake [10]. Support for vaccination was higher for older adults than for young children, suggesting that participants did not hold a uniform attitude toward COVID-19 vaccination across target groups. Rather, their support appeared to vary according to the perceived benefit-risk rationale for each recipient group. This pattern is consistent with the clearer risk-based rationale for vaccinating older adults given that older age is a major risk factor for severe COVID-19 outcomes [22]. Previous studies have also documented population-specific determinants of vaccination attitudes among older adults and parents of young children [23,24]. Support for COVID-19 vaccination may therefore depend not only on participants’ own vaccine confidence but also on how clearly the rationale is communicated for each target population.

The strong association between unwillingness to receive influenza vaccination and hesitancy toward future annual COVID-19 vaccination provides additional insight. Influenza vaccination is an established example of periodic respiratory-virus vaccination for health care personnel [25,26]. The observed association may therefore reflect broader attitudes toward repeated vaccination rather than attitudes toward COVID-19 vaccination alone. However, this comparison should be interpreted cautiously. COVID-19 vaccination and influenza vaccination differ in their history, policy context, perceived urgency, and public communication environment. Therefore, influenza vaccination willingness should be viewed as a useful comparative reference for periodic vaccination attitudes, not as a direct equivalent of future COVID-19 vaccination willingness.

These findings suggest that future vaccination strategies for physicians and medical trainees should move beyond general promotional messages and emphasize transparent, regularly updated, and clinically interpretable evidence on vaccine effectiveness, safety, durability of protection, variant relevance, and benefit-risk profiles after prior vaccination and SARS-CoV-2 infection. Hospitals and professional organizations could incorporate such evidence into continuing medical education and develop target population–specific communication materials to support discussions with older adults and parents of young children. Prospective studies are needed to evaluate whether these approaches improve vaccination confidence and subsequent vaccination behavior.

This study has several strengths, including its large multicenter sample across 7 geographic regions of China and its assessment of both participants’ own vaccination willingness and their support for vaccination among older adults and young children. However, several limitations should be considered. First, the cross-sectional design precludes causal inference and cannot capture changes in vaccination attitudes over time. Second, participants were recruited from public hospitals using predefined recruitment targets rather than probability sampling and were limited to physicians and medical trainees, which may restrict the generalizability of the findings. Because the questionnaire was distributed through direct invitations and department-level WeChat groups, the response rate could not be calculated, and selection bias remains possible. Third, willingness to receive vaccination and willingness to support vaccination for older adults and young children were self-reported and may not reflect actual future behavior or real-world clinical recommendation practices. Fourth, sex and income data were not collected, preventing assessment of these factors. Fifth, because reasons for hesitancy toward COVID-19 and influenza vaccination were assessed using vaccine-specific response options, comparisons between the two vaccines should be interpreted descriptively rather than as direct item-by-item comparisons.

In conclusion, high prior COVID-19 vaccination uptake did not translate into similarly high willingness to receive future annual vaccination among physicians and medical trainees. Future vaccination strategies should address concerns about the evidence supporting repeated vaccination and the differing levels of support observed for older adults and young children.

## Supplementary material

10.2196/88931 Multimedia Appendix 1 Questionnaire for the COVID-19 awareness and vaccination attitudes survey distributed to physicians and medical trainees.

10.2196/88931 Multimedia Appendix 2 Informed consent form.

10.2196/88931 Multimedia Appendix 3 Detailed breakdowns of vaccination status, willingness, and vaccine types, Human Development Index classification, hospital classification, clinical department classification, and univariable and multivariable modified Poisson regression analyses.

## Abbreviations

aPR: adjusted prevalence ratio
CVR: content validity ratio
HDI: Human Development Index
PR: prevalence ratio
