# Supplementary material for: Postpandemic COVID-19 Vaccination Willingness Among Physicians and Medical Trainees in China: Cross-Sectional Study
Source: JMIR Public Health Surveill. 2026 Oct 6;12:e88931. doi: 10.2196/88931 (PMC13641312; doi:10.2196/88931)
Supplement: Supplementary file 1 [file publichealth-v12-e88931-s001.pdf]

COVID-19 Awareness and Vaccination Attitudes Survey Among Physicians and Medical Trainees

English Translation of the Original Chinese Questionnaire

This survey, initiated by the China Primary Health Care Foundation and the National Center for Respiratory Medicine, aims to understand physicians' and medical trainees' awareness of COVID-19 and attitudes toward COVID-19 vaccination. We sincerely invite you to participate.

This questionnaire is confidential and is expected to take approximately 10 minutes. Responses used for analysis will be de-identified. A mobile phone number was collected solely for distributing the CNY 50 participant compensation and was not included in the analytic dataset.

Research objectives:

1. To assess awareness of COVID-19 among physicians and medical trainees.

2. To assess awareness of and attitudes toward vaccination among physicians and medical trainees.

Basic Information

The city where you work permanently: [Fill in the blank] *

____

Your hospital: [Single-choice question] *

- China-Japan Friendship Hospital
- Henan Provincial People's Hospital
- Ruijin Hospital, Shanghai Jiaotong University School of Medicine
- Qingdao Municipal Hospital
- Sichuan Provincial People's Hospital
- Xiangya Hospital, Central South University
- The First Affiliated Hospital of Xi'an Jiaotong University
- The Second Affiliated Hospital of Harbin Medical University
- Wuhan Central Hospital
- Yichang Central Hospital
- Fuyang Second People's Hospital
- Third People's Hospital of Chengdu
- Fujian Provincial Hospital
- Longgang Central Hospital, Shenzhen
- Second People's Hospital of Lanzhou
- Xi'an Chest Hospital
- Capital Medical University Affiliated Beijing Luhe Hospital
- Beijing Chuiyangliu Hospital
- Longquanyi District First People's Hospital, Chengdu
- The Sixth People's Hospital of Shenyang
- Dongsheng District People's Hospital

Personal department or major studied: [Single-choice question] *

- Internal medicine (for departments without subspecialty classification)
- Surgery (for departments without subspecialty classification)
- General practice (for departments without subspecialty classification)
- Department of Respiratory and Critical Care Medicine
- Cardiology
- Gastroenterology
- Rheumatology and Immunology
- Neurology
- Pediatrics
- Emergency Department
- Department of Infectious Diseases
- Oncology
- Hematology
- Nephrology
- Cardiac Surgery
- Thoracic Surgery
- Pain Department
- General Surgery
- Orthopedics
- Urology
- Plastic Surgery
- Rehabilitation Department
- Ophthalmology
- Otolaryngology, Head and Neck Surgery
- Dentistry
- Dermatology
- Department of Anesthesiology
- Department of Critical Care Medicine
- Obstetrics and Gynecology
- Radiology Department
- Laboratory Department
- Blood Transfusion Department
- Ultrasound Department
- Others

Other departments: [Fill in the blank]

____

Display logic: This item was displayed only if "Others" was selected for department or major.

Your job title: [Single-choice question] *

- Medical trainee before standardized residency training
- Resident doctor (including standardized residency trainees)
- Attending physician
- Associate chief physician
- Chief physician

Your age group: [Single-choice question] *

- 20-29 years
- 30-39 years
- 40-49 years
- 50-59 years
- 60 years or older

Have you ever treated patients with COVID-19, such as in designated hospitals? [Single-choice question] *****

- Yes
- No

Have you ever been infected with SARS-CoV-2? [Single-choice question] *****

- Yes
- No

Your education level: [Single-choice question] *****

- Junior high school or below
- High school or vocational high school
- College or undergraduate degree
- Master's degree
- Doctoral degree

COVID-19 Awareness

This section includes questions on:

1. Perceived pathogenicity and infectiousness of COVID-19;

2. Identification of groups at high risk for severe or critical COVID-19;

3. Awareness of COVID-19 vaccination.

1. Which of the following diseases do you think is similar to the current COVID-19 in pathogenicity for the general population? [Single-choice question] *****

- Influenza
- Common cold
- SARS
- Pneumonia
- Plague
- Unknown

2. Which of the following diseases do you think is similar to the Omicron variant of SARS-CoV-2 in contagiousness? [Single-choice question] *****

- Influenza (R0 = 1-2)
- Smallpox (R0 = 3.5-6)
- Varicella (R0 = 9-12)
- Tuberculosis (R0 < 1)
- Measles (R0 approximately 18)
- Unknown

3. Who do you think are the high-risk groups for developing severe COVID-19? [Multiple-choice question] *

- Children
- Older adults aged over 65 years
- People with underlying diseases, such as cardiovascular and cerebrovascular diseases, chronic lung diseases, diabetes, chronic liver or kidney diseases, and tumors
- People with obesity (BMI >= 30)
- People receiving immunosuppressive therapy
- Pregnant and perinatal women
- Smokers without underlying diseases such as COPD
- Healthy people who have not been vaccinated

Vaccine Awareness and Vaccination Attitudes

1. Have you received a COVID-19 vaccine? [Single-choice question] *

- Not vaccinated (skip manufacturer and dose questions)
- Already vaccinated and clearly remember the vaccine manufacturer
- Already vaccinated but do not remember the vaccine manufacturer (skip manufacturer-specific dose questions)

1.1 How many doses of inactivated vaccine have you received (Beijing Biotech, Sinovac, Wuhan Biotech, or others)? [Single-choice question] *

- 1 dose
- 2 doses
- 3 doses
- 4 doses or more
- 0 doses

1.2 How many doses of recombinant protein vaccine have you received (Anhui Zhifei or others)? [Single-choice question] *

- 1 dose
- 2 doses
- 3 doses
- 4 doses or more
- 0 doses

1.3 How many doses of mRNA vaccine have you received (BioNTech/Pfizer/Fosun, Moderna, or others)? [Single-choice question] *

- 1 dose
- 2 doses
- 3 doses
- 4 doses or more
- 0 doses

1.4 How many doses of adenovirus-vectored vaccine have you received (CanSino or others)? [Single-choice question] *

- 1 dose
- 2 doses

- 3 doses
- 4 doses or more
- 0 doses

1.5 How many times have you received the inhaled recombinant COVID-19 vaccine? [Single-choice question] *

- 1 time
- 2 times
- 3 times
- 4 times or more
- 0 times

Display logic: Participants who completed this item skipped to the subsequent vaccine attitude questions.

1.6 How many total doses of COVID-19 vaccine have you received? [Single-choice question] *

- 1 dose
- 2 doses
- 3 doses
- 4 doses or more
- 0 doses

1.7 When was the last time you received a COVID-19 vaccine? [Fill in the blank] *

____

2. Would you proactively receive COVID-19 vaccination every year in the future? (Similar to influenza vaccination) [Single-choice question] *

- Yes
- No

What are the main reasons why you are unwilling to receive future COVID-19 vaccination? [Multiple-choice question] *

- Insufficient disclosure of domestic vaccine clinical trial data
- I do not want to be vaccinated because I think the protective effectiveness of COVID-19 vaccines is not ideal
- Concerns about the safety of COVID-19 vaccines, even if sufficient data are disclosed and the safety profile is good
- I think I am in good health and do not need to worry
- I have underlying diseases and am afraid to receive COVID-19 vaccination
- Fear of injections
- Others

Display logic: This item was displayed only if the participant answered "No" to future annual COVID-19 vaccination.

If a nasal spray or inhaled vaccine were available, would you be willing to receive it? [Single-choice question] *

- Yes
- No

3. Would you proactively receive influenza vaccination? [Single-choice question] *

- Yes
- No

Reasons for not wanting to receive influenza vaccination: [Multiple-choice question] *****

- Concerns about the safety of influenza vaccines
- I think the protective effectiveness of influenza vaccines is not ideal
- I think I am in good health and do not need to worry about influenza
- I do not know where to receive influenza vaccination
- I do not have time to receive influenza vaccination
- Influenza vaccination requires out-of-pocket payment
- I have underlying diseases and am afraid to receive influenza vaccination
- I want to be vaccinated, but the community is out of stock
- Others

Display logic: This item was displayed only if the participant answered "No" to influenza vaccination.

4. Which COVID-19 vaccine would you choose first? [Single-choice question] *****

- Inactivated vaccine
- Recombinant protein vaccine
- mRNA vaccine
- Adenovirus-vectored vaccine (injection)
- I do not want to be vaccinated
- Inhaled vaccine

5. Do you agree with using compulsory measures to promote vaccination, such as restricting access to public places? [Single-choice question] *****

- Yes
- No

6. Do you agree that people who have not been vaccinated should bear the risk of severe disease themselves and that medical insurance should not pay for the related costs? (This refers to a policy approach used in Singapore; agreeing to payment may lead to pressure on universal medical insurance funds.) [Single-choice question] *****

- Agree
- Disagree

7. What is the maximum amount of money you would accept for annual vaccination (influenza or COVID-19)? [Single-choice question] *****

- CNY 10-50
- CNY 50-100
- CNY 100-150
- CNY 150-200
- CNY 200-300
- CNY 300 or above

8. If older adults in your family aged over 65 years have no clear contraindications, would you support their COVID-19 vaccination? [Single-choice question] *****

- Yes
- No

9. If there are children younger than 6 years in your family, would you support their COVID-19 vaccination? [Single-choice question] *

- Yes
- No

10. How long after SARS-CoV-2 infection should COVID-19 vaccination be considered to boost immunity? [Single-choice question] *

- 1 month after infection
- 3 months after infection
- 6 months after infection
- 1 year after infection
- Do not vaccinate because reinfection symptoms are milder
- Do not want to be vaccinated

11. Please assess the risk of SARS-CoV-2 reinfection in the following situations: [Matrix scale question] *

Situation	High risk	Medium risk	Low risk
Contact with patients with COVID-19 without wearing a mask 1 month after recovery	○	○	○
Contact with patients with COVID-19 without wearing a mask 1 year after recovery	○	○	○
The local SARS-CoV-2 epidemic strain mutates and produces immune escape	○	○	○

12. What do you think about post-COVID-19 condition or sequelae after SARS-CoV-2 infection? [Multiple-choice question] *

- There is no risk of post-COVID-19 sequelae
- The original strains in the early stage were highly pathogenic and had obvious sequelae; current mutant strains basically do not cause post-COVID-19 sequelae
- SARS-CoV-2 infection can lead to post-COVID-19 sequelae, but it does not significantly affect quality of life
- Only patients with severe illness are at risk of post-COVID-19 sequelae
- Even mild cases may lead to post-COVID-19 sequelae and affect quality of life

Please enter your mobile phone number, used only to distribute participant compensation (CNY 50): [Fill in the blank] *

____
