# Supplementary material for: Postpandemic COVID-19 Vaccination Willingness Among Physicians and Medical Trainees in China: Cross-Sectional Study
Source: JMIR Public Health Surveill. 2026 Oct 6;12:e88931. doi: 10.2196/88931 (PMC13641312; doi:10.2196/88931)
Supplement: Supplementary file 2 [file publichealth-v12-e88931-s002.pdf]

Informed Consent

English Translation of the Original Chinese Electronic Informed Consent Form

Principal Investigator

Name: Bin Cao

Institution: National Center for Respiratory Medicine, China-Japan Friendship Hospital

Email: caobin_ben@163.com

Purpose

You are invited to participate in a research study. The purpose of this study is to assess physicians' and medical trainees' awareness of COVID-19, attitudes toward COVID-19 vaccination, willingness to receive future COVID-19 vaccination, and willingness to support COVID-19 vaccination for older adults and young children.

Procedures

This study will be conducted through an online questionnaire. You will be asked to provide basic demographic and professional information and answer questions about COVID-19 awareness, vaccination history, and vaccination attitudes. The questionnaire will take approximately 10 minutes to complete.

Confidentiality

Your responses will be kept confidential. Information used for analysis will be de-identified. A mobile phone number was collected solely for distributing the CNY 50 participant compensation and was not included in the analytic dataset. Survey data will be stored in a password-protected database accessible only to authorized study investigators.

Voluntary Participation

Your participation is voluntary. You may choose not to participate or withdraw at any time without giving a reason. Refusal to participate or withdrawal from the study will not affect your relationship with the investigators or your institution.

Contact Information

If you have questions about this study, please contact the study investigator using the contact information above. If you have questions about your rights as a research participant, please contact Yeming Wang at 18810663558, China-Japan Friendship Hospital.

Electronic Consent

I have read and understood the information above and have had the opportunity to ask questions. My participation is voluntary, and I may withdraw at any time without giving a reason.

If you agree to participate, please click "Agree" to begin the questionnaire. If you do not agree, please click "Disagree" to exit the survey.
