# Supplementary material for: Postpandemic COVID-19 Vaccination Willingness Among Physicians and Medical Trainees in China: Cross-Sectional Study
Source: JMIR Public Health Surveill. 2026 Oct 6;12:e88931. doi: 10.2196/88931 (PMC13641312; doi:10.2196/88931)
Supplement: Supplementary file 3 [file publichealth-v12-e88931-s003.docx]

**Supplementary Materials**

**Figure S1. Prior COVID-19 vaccination status, willingness to receive future annual COVID-19 vaccination, and COVID-19 vaccine types among physicians and medical trainees.**

The left panels show prior COVID-19 vaccination status and willingness to receive future annual COVID-19 vaccination among participants. The upper-right panel shows previously received COVID-19 vaccine types among participants who had received at least 1 dose; each vaccine type was assessed separately, and participants could have received more than one type, so percentages may sum to more than 100%. The lower-right panel shows preferred vaccine types among participants willing to receive future annual COVID-19 vaccination; participants who did not wish to receive any of the vaccine types shown are not displayed, so the percentages sum to less than 100%.

**Table S1. Human Development Index classification of participating regions**

| HDI level | Percentile of HDI | HDI value | Included provinces, municipalities, or autonomous regions |
| --- | --- | --- | --- |
| Low HDI | ≤25th | ≤0.708 | Anhui, Sichuan, Gansu |
| Middle HDI | >25th to 75th | >0.708 to 0.754 | Hubei, Shandong, Hunan, Fujian, Shaanxi, Inner Mongolia, Henan, Heilongjiang |
| High HDI | >75th | >0.754 | Guangdong, Liaoning, Beijing, Shanghai |

Note. HDI: Human Development Index. HDI is a composite measure of socioeconomic development including life expectancy, education, and gross income per capita. Classification was based on the HDI distribution of participating regions.

**Table S2. Hospital classification of participating hospitals**

| Hospital classification | Participating hospitals |
| --- | --- |
| Top Grade A tertiary hospital | China-Japan Friendship Hospital; Henan Provincial People's Hospital; Ruijin Hospital, Shanghai Jiaotong University School of Medicine; Qingdao Municipal Hospital; Sichuan Provincial People's Hospital; Xiangya Hospital, Central South University; The First Affiliated Hospital of Xi'an Jiaotong University; The Second Affiliated Hospital of Harbin Medical University |
| Other Grade A tertiary hospital | Wuhan Central Hospital; Yichang Central Hospital; Fuyang Second People's Hospital; Third People's Hospital of Chengdu; Fujian Provincial Hospital; Longgang Central Hospital, Shenzhen; Second People's Hospital of Lanzhou; Xi'an Chest Hospital |
| Non-tertiary or other hospital | Capital Medical University Affiliated Beijing Luhe Hospital; Beijing Chuiyangliu Hospital; Longquanyi District First People's Hospital, Chengdu; The Sixth People's Hospital of Shenyang; Dongsheng District People's Hospital |

Note. Hospital classification was based on China's hospital classification system and prespecified hospital ranking criteria, including the China Hospital Reputation Rankings provided by Fudan University Hospital Management Institute and the 2018-2022 overall science and technology evaluation metrics rankings.

**Table S3. Clinical department classification**

| Clinical department classification | Clinical departments |
| --- | --- |
| COVID-19-related internal medicine departments | Pulmonary and Critical Care Medicine (PCCM); Emergency Medicine Department; Infectious Diseases Department; Critical Care Medicine Department |
| Other internal medicine departments | Internal Medicine (applicable to undivided subspecialties); Cardiology Department; Gastroenterology Department; Rheumatology and Immunology Department; Neurology Department; Oncology Department; Hematology Department; Nephrology Department |
| Surgical departments | Surgery (applicable to undivided subspecialties); Cardiovascular Surgery; Thoracic Surgery; Pain Management Department; General Surgery; Orthopedic Surgery; Urology Department; Plastic Surgery; Ophthalmology Department; Otorhinolaryngology (ENT) Head and Neck Surgery; Oral and Maxillofacial Surgery; Dermatology Department; Anesthesiology Department; Obstetrics and Gynecology Department |
| Other departments | General Practice (applicable to undivided subspecialties); Pediatrics Department; Rehabilitation Medicine Department; Radiology Department; Clinical Laboratory (Pathology) Department; Blood Transfusion Department; Ultrasonography Department; Others |

**Table S4A. Univariable and multivariable modified Poisson regression analyses of factors associated with hesitancy toward future annual COVID-19 vaccination**

| **Variable** | **Category** | **Unadjusted PR (95% CI)** | **P value** | **Adjusted PR (95% CI)** | **P value** |
| --- | --- | --- | --- | --- | --- |
| Age group | 20-29 y | Reference |  | Reference |  |
|  | 30-39 y | 0.825 (0.749-0.908) | <.001 | 0.927 (0.825-1.041) | .200 |
|  | 40-49 y | 0.887 (0.782-1.005) | .060 | 0.997 (0.840-1.185) | .977 |
|  | ≥50 y | 0.873 (0.723-1.054) | .157 | 1.006 (0.802-1.261) | .960 |
| Education level | College or lower | Reference |  | Reference |  |
|  | Master's degree | 1.313 (1.193-1.445) | <.001 | 1.111 (1.023-1.206) | .013 |
|  | Doctoral degree | 1.499 (1.318-1.706) | <.001 | 1.203 (1.068-1.355) | .002 |
| HDI level | High HDI | Reference |  | Reference |  |
|  | Middle HDI | 0.973 (0.883-1.072) | .575 | 0.912 (0.831-1.001) | .052 |
|  | Low HDI | 0.835 (0.740-0.943) | .004 | 1.001 (0.895-1.121) | .980 |
| Hospital classification | Top Grade A tertiary hospital | Reference |  | Reference |  |
|  | Other Grade A tertiary hospital | 0.787 (0.715-0.866) | <.001 | 0.890 (0.813-0.974) | .011 |
|  | Non-tertiary or other hospital | 0.749 (0.669-0.840) | <.001 | 0.808 (0.724-0.901) | <.001 |
| Career stage | Medical trainee | Reference |  | Reference |  |
|  | Resident | 1.170 (0.996-1.374) | .056 | 1.078 (0.936-1.241) | .296 |
|  | Attending physician | 0.986 (0.833-1.166) | .866 | 1.074 (0.899-1.282) | .432 |
|  | Associate chief/chief physician | 1.025 (0.861-1.221) | .781 | 0.962 (0.780-1.186) | .716 |
| Experience treating patients with COVID-19 | No | Reference |  | Reference |  |
|  | Yes | 0.909 (0.820-1.008) | .071 | 1.039 (0.943-1.145) | .439 |
| Prior COVID-19 vaccination | Yes | Reference |  | Reference |  |
|  | No | 1.605 (1.368-1.882) | <.001 | 1.272 (1.110-1.458) | <.001 |
| Willingness to receive influenza vaccination | Yes | Reference |  | Reference |  |
|  | No | 4.188 (3.785-4.632) | <.001 | 4.109 (3.712-4.549) | <.001 |
| Clinical department | COVID-19-related internal medicine departments | Reference |  | Reference |  |
|  | Other internal medicine departments | 1.007 (0.911-1.114) | .889 | 0.908 (0.835-0.988) | .025 |
|  | Surgical departments | 1.120 (0.983-1.276) | .088 | 1.029 (0.921-1.149) | .619 |
|  | Other departments | 0.964 (0.838-1.108) | .603 | 0.993 (0.881-1.119) | .908 |
| SARS-CoV-2 infection history | No | Reference |  | Reference |  |
|  | Yes | 1.097 (0.916-1.315) | .314 | 1.063 (0.922-1.226) | .400 |

Note. PR: prevalence ratio; HDI: Human Development Index; SARS-CoV-2: severe acute respiratory syndrome coronavirus 2. Unadjusted PRs were estimated using univariable modified Poisson regression models. Adjusted PRs were estimated using multivariable modified Poisson regression models with robust standard errors. P values <.001 are shown as <.001; all other P values are rounded to 3 decimal places.

**Table S4B. Univariable and multivariable modified Poisson regression analyses of factors associated with hesitancy to support COVID-19 vaccination for older adults**

| **Variable** | **Category** | **Unadjusted PR (95% CI)** | **P value** | **Adjusted PR (95% CI)** | **P value** |
| --- | --- | --- | --- | --- | --- |
| Age group | 20-29 y | Reference |  | Reference |  |
|  | 30-39 y | 0.563 (0.441-0.719) | <.001 | 0.931 (0.654-1.324) | .690 |
|  | 40-49 y | 0.725 (0.532-0.987) | .041 | 1.378 (0.811-2.342) | .235 |
|  | ≥50 y | 0.884 (0.580-1.348) | .568 | 1.653 (0.856-3.190) | .134 |
| Education level | College or lower | Reference |  | Reference |  |
|  | Master's degree | 1.108 (0.882-1.391) | .380 | 0.969 (0.769-1.221) | .790 |
|  | Doctoral degree | 1.252 (0.896-1.751) | .188 | 0.979 (0.683-1.405) | .910 |
| HDI level | High HDI | Reference |  | Reference |  |
|  | Middle HDI | 0.886 (0.700-1.121) | .314 | 0.936 (0.724-1.209) | .613 |
|  | Low HDI | 0.652 (0.480-0.886) | .006 | 0.803 (0.581-1.111) | .185 |
| Hospital classification | Top Grade A tertiary hospital | Reference |  | Reference |  |
|  | Other Grade A tertiary hospital | 0.626 (0.486-0.806) | <.001 | 0.846 (0.639-1.120) | .243 |
|  | Non-tertiary or other hospital | 0.871 (0.673-1.128) | .295 | 1.079 (0.798-1.459) | .620 |
| Career stage | Medical trainee | Reference |  | Reference |  |
|  | Resident | 0.890 (0.642-1.233) | .484 | 0.845 (0.596-1.198) | .344 |
|  | Attending physician | 0.513 (0.356-0.739) | <.001 | 0.572 (0.351-0.934) | .026 |
|  | Associate chief/chief physician | 0.610 (0.418-0.891) | .011 | 0.482 (0.261-0.892) | .020 |
| Experience treating patients with COVID-19 | No | Reference |  | Reference |  |
|  | Yes | 0.679 (0.535-0.861) | .001 | 0.994 (0.759-1.302) | .966 |
| Prior COVID-19 vaccination | Yes | Reference |  | Reference |  |
|  | No | 1.865 (1.196-2.907) | .006 | 1.421 (0.942-2.145) | .094 |
| Own hesitancy toward future annual COVID-19 vaccination | No | Reference |  | Reference |  |
|  | Yes | 5.365 (4.140-6.953) | <.001 | 5.125 (3.945-6.658) | <.001 |
| Clinical department | COVID-19-related internal medicine departments | Reference |  | Reference |  |
|  | Other internal medicine departments | 1.473 (1.125-1.930) | .005 | 1.363 (1.046-1.776) | .022 |
|  | Surgical departments | 2.037 (1.484-2.796) | <.001 | 1.692 (1.237-2.314) | .001 |
|  | Other departments | 1.431 (1.008-2.032) | .045 | 1.405 (1.000-1.973) | .050 |
| SARS-CoV-2 infection history | No | Reference |  | Reference |  |
|  | Yes | 0.710 (0.497-1.014) | .060 | 0.706 (0.507-0.984) | .040 |

Note. PR: prevalence ratio; HDI: Human Development Index; SARS-CoV-2: severe acute respiratory syndrome coronavirus 2. Unadjusted PRs were estimated using univariable modified Poisson regression models. Adjusted PRs were estimated using multivariable modified Poisson regression models with robust standard errors. P values <.001 are shown as <.001; all other P values are rounded to 3 decimal places.

**Table S4C. Univariable and multivariable modified Poisson regression analyses of factors associated with hesitancy to support COVID-19 vaccination for young children**

| **Variable** | **Category** | **Unadjusted PR (95% CI)** | **P value** | **Adjusted PR (95% CI)** | **P value** |
| --- | --- | --- | --- | --- | --- |
| Age group | 20-29 y | Reference |  | Reference |  |
|  | 30-39 y | 0.987 (0.860-1.132) | .847 | 1.193 (0.991-1.436) | .062 |
|  | 40-49 y | 1.248 (1.061-1.469) | .007 | 1.387 (1.072-1.794) | .013 |
|  | ≥50 y | 1.437 (1.162-1.777) | <.001 | 1.524 (1.126-2.063) | .006 |
| Education level | College or lower | Reference |  | Reference |  |
|  | Master's degree | 1.218 (1.073-1.383) | .002 | 1.073 (0.946-1.217) | .273 |
|  | Doctoral degree | 1.411 (1.181-1.685) | <.001 | 0.999 (0.829-1.204) | .990 |
| HDI level | High HDI | Reference |  | Reference |  |
|  | Middle HDI | 0.801 (0.706-0.909) | <.001 | 0.818 (0.712-0.938) | .004 |
|  | Low HDI | 0.646 (0.548-0.761) | <.001 | 0.746 (0.627-0.887) | <.001 |
| Hospital classification | Top Grade A tertiary hospital | Reference |  | Reference |  |
|  | Other Grade A tertiary hospital | 0.831 (0.726-0.951) | .007 | 0.958 (0.828-1.108) | .562 |
|  | Non-tertiary or other hospital | 0.988 (0.856-1.141) | .873 | 0.997 (0.848-1.173) | .974 |
| Career stage | Medical trainee | Reference |  | Reference |  |
|  | Resident | 1.252 (0.990-1.585) | .061 | 1.130 (0.889-1.437) | .317 |
|  | Attending physician | 1.089 (0.854-1.388) | .493 | 0.949 (0.713-1.263) | .720 |
|  | Associate chief/chief physician | 1.459 (1.146-1.859) | .002 | 1.110 (0.803-1.534) | .528 |
| Experience treating patients with COVID-19 | No | Reference |  | Reference |  |
|  | Yes | 0.935 (0.810-1.078) | .354 | 0.927 (0.796-1.081) | .333 |
| Prior COVID-19 vaccination | Yes | Reference |  | Reference |  |
|  | No | 1.896 (1.534-2.344) | <.001 | 1.423 (1.163-1.740) | <.001 |
| Own hesitancy toward future annual COVID-19 vaccination | No | Reference |  | Reference |  |
|  | Yes | 2.975 (2.629-3.366) | <.001 | 2.882 (2.544-3.265) | <.001 |
| Clinical department | COVID-19-related internal medicine departments | Reference |  | Reference |  |
|  | Other internal medicine departments | 1.070 (0.935-1.225) | .323 | 1.076 (0.947-1.223) | .263 |
|  | Surgical departments | 1.208 (1.016-1.437) | .033 | 1.181 (0.997-1.398) | .054 |
|  | Other departments | 0.819 (0.667-1.006) | .057 | 0.855 (0.705-1.038) | .113 |
| SARS-CoV-2 infection history | No | Reference |  | Reference |  |
|  | Yes | 0.849 (0.690-1.045) | .123 | 0.816 (0.670-0.992) | .041 |

Note. PR: prevalence ratio; HDI: Human Development Index; SARS-CoV-2: severe acute respiratory syndrome coronavirus 2. Unadjusted PRs were estimated using univariable modified Poisson regression models. Adjusted PRs were estimated using multivariable modified Poisson regression models with robust standard errors. P values <.001 are shown as <.001; all other P values are rounded to 3 decimal places.
